# Clinicopathological cohort study of kidney biopsy findings resulting in dialysis during long-term follow-up exceeding 30 years

**DOI:** 10.1007/s10157-025-02706-8

**Published:** 2025-05-28

**Authors:** Yoichi Oshima, Naoki Sawa, Masayuki Yamanouchi, Akinari Sekine, Hiroki Mizuno, Daisuke Ikuma, Yuki Oba, Noriko Inoue, Kiho Tanaka, Eiko Hasegawa, Tatsuya Suwabe, Kei Kono, Keiichi Kinowaki, Kenichi Ohashi, Yutaka Yamaguchi, Junichi Hoshino, Yoshifumi Ubara

**Affiliations:** 1 Nephrology Center and Okinaka Memorial Institute for Medical Research, Toranomon Hospital, Tokyo, Japan; 2 Department of Nephrology, Tokyo Saiseikai Central Hospital, Tokyo, Japan; 3 Department of Pathology, Toranomon Hospital, Tokyo, Japan; 4 Department of Human Pathology, Institute of Science Tokyo, Tokyo, Japan; 5 Yamaguchi’s Pathology Laboratory, Chiba, Japan; 6 Nephrology Center, Toranomon Hospital Kajigaya, 1-3-1, Kajigaya, Kawasaki, Kanagawa 213-8587 Japan; 7 Department of Nephrology, Tokyo Women’s Medical University, Tokyo, Japan

**Keywords:** Benign nephrosclerosis, IgA nephropathy, Diabetic nephropathy, Kidney biopsy, End stage kidney disease

## Abstract

**Background:**

Numerous kidney diseases progress to end-stage kidney disease (ESKD); however, a limited number of cohort studies have evaluated the underlying kidney diseases through kidney biopsy (KB).

**Methods:**

We retrospectively evaluated all patients who initiated dialysis at Toranomon Hospital, Japan, from 1985 to 2019, and whose underlying kidney disease had been diagnosed by KB. The data on histopathological diagnosis and various clinical characteristics were collected and analyzed for 357 patients.

**Results:**

The most prevalent underlying diseases, which constituted the primary endpoint of this study, were diabetic nephropathy (DN; *n* = 100, 28.0%), IgA nephropathy (IgAN; *n* = 99, 27.7%), and focal segmental glomerulosclerosis (*n* = 34, 9.5%). Benign nephrosclerosis (BNS; *n* = 1, 0.3%), that is, arteriosclerosis/arteriolosclerosis without distinct glomerulopathy, was rare. As the secondary endpoint, Cox regression analysis revealed that lower eGFR (*p* < 0.0001), higher proteinuria (*p* < 0.0001), older age (*p* = 0.005) and presence of DN (*p* = 0.008) were significant independent risk factors for early dialysis initiation. In the subgroup analysis, when comparing DN and IgAN, significantly earlier dialysis initiation was observed in DN than in IgAN by log-rank analysis (*p* < 0.0001), as well as after adjustment for baseline clinical characteristics using propensity score matching (*n* = 45 each) (*p* = 0.023).

**Conclusions:**

We identified a list of kidney diseases that were at risk for ESKD at the time of KB through a long-term follow-up. DN and IgAN are the two primary causes of ESKD, whereas BNS is an infrequent direct cause of ESKD in patients requiring kidney biopsy.

**Supplementary Information:**

The online version contains supplementary material available at 10.1007/s10157-025-02706-8.

## Introduction

The Japanese Society for Dialysis Therapy publishes an annual report presenting the results of a questionnaire survey conducted at dialysis facilities. A 2018 report identified diabetic nephropathy, chronic glomerulonephritis, and nephrosclerosis as the three primary cause of dialysis in Japan and noted an increasing prevalence of dialysis among older adults [1]. In this report, patients with a history of diabetes mellitus (DM) were classified as having diabetic nephropathy (DN); those with hematuria and/or nephrotic-range proteinuria were classified as having chronic glomerulonephritis; and those with hypertension accompanied by minimal proteinuria and atrophic kidneys were classified as having benign nephrosclerosis (BNS), alternatively termed arteriosclerosis/arteriolosclerosis without glomerulopathy. The United States Renal Data System, which compiles the causes of end-stage kidney disease (ESKD), reported in 2012 that underlying kidney diseases included diabetes (38%) and hypertension-related nephropathy (25%) [2]. However, it is important to note that the results of both the Japanese and US reports were based on the subjective assessments of the attending physicians rather than on kidney biopsy findings.

Studies based on the kidney biopsies have yielded significantly different results from those described above. A retrospective cohort study conducted in the UK, which included 463 patients aged > 70 years who underwent kidney biopsy, reported that vasculitis and paraproteinemia were the most prevalent diseases leading to dialysis, whereas primary glomerulonephritis was the least common [3]. This report excluded diabetic nephropathy, possibly because of the UK-specific criteria for kidney biopsy [3].

There is a paucity of studies utilizing histopathological evaluations of kidney biopsies to determine the most common underlying diseases resulting in dialysis initiation. Consequently, we conducted a retrospective cohort study of patients who underwent kidney biopsy and subsequently progressed to ESKD requiring dialysis initiation. Owing to the rigorous development of guidelines in recent years, the trend was observed by dividing the cohort into three groups according to the time periods and analyzed. Additionally, we analyzed and described the baseline clinical characteristics at the time of kidney biopsy and investigated independent risk factors associated with early dialysis initiation using multivariate Cox regression analysis. Furthermore, we analyzed the clinical characteristics of the two major causes of dialysis initiation, DN and IgAN, and compared the two groups.

## Methods

### Patients

In this retrospective, single-center cohort study, we examined 1382 patients who initiated dialysis and underwent kidney biopsy at our nephrology center at Toranomon Hospital, Tokyo, Japan, between January 1985 and December 2019. Patients with DM were predominantly referred to by diabetologists, whereas nondiabetic patients were referred to by general physicians or non-nephrologists. The investigators noted that all patients were of Japanese ethnicity. Our primary endpoint was to compile the underlying kidney diseases leading to ESKD, identified by kidney biopsy. The secondary endpoint was to analyze the progressive risk factors associated with a shorter time to ESKD. Subgroup analysis encompassed the following: underlying kidney diseases across the three periods, underlying kidney diseases in each age group at dialysis initiation, clinical characteristics of patients with DN among the three periods, clinical characteristics of patients with IgAN among the three periods, and comparison between DN and IgAN.

At our institution, kidney biopsies are considered for all patients upon referral. The decision to perform a kidney biopsy was made by the chief physician and nephrologist team based on the following criteria: proteinuria (≥ 0.2 g/day), hematuria (> 5 red blood cells/high-power field [HPF]), or renal dysfunction (estimated glomerular filtration rate [eGFR], < 60 mL/min/1.73m^2^). These criteria are applied to patients regardless of their DM status. In clinical practice, kidney biopsy is not performed in numerous patients, even if they meet the diagnostic criteria; for instance, patients with severe physical deformity may be unable to assume the prone position required for kidney biopsy.

### Clinical data and pathological diagnosis of kidney biopsy

The following clinical data were collected from the medical records at the time of kidney biopsy: histopathological diagnosis; age; sex; and blood tests results, including serum creatinine (Cre; mg/dL) and estimated glomerular filtration rate (eGFR; calculated from Cre; mL/min/1.73m^2^), which were measured with a previously reported method [4]. Urine tests included assessment of proteinuria and hematuria (red blood cells per HPF in resuspended sediment, categorized as follows: grade 0 [< 1 red blood cell], grade 1 [1–5 red blood cells], grade 2 [6–10 red blood cells], grade 3 [11–30 red blood cells], grade 4 [> 30 red blood cells]). In addition, the data were collected on the number of days between kidney biopsy and dialysis initiation, as well as age at dialysis initiation.

Kidney biopsy specimens were analyzed using light microscopy (LM), immunofluorescence microscopy (IF), and electron microscopy (EM) using standard methodologies [5]. Kidney biopsy findings were evaluated by four pathologists. To validate the current pathological diagnosis, the specimens were re-evaluated according to the most recent pathological criteria. Concise diagnostic characteristics of the respective kidney diseases and other methods are described in the supplementary material.

The Toranomon Hospital Ethics Committee reviewed and approved the protocol (approval code 2081-B), and informed consent was obtained in the form of an opt-out option on the website. Those who opted out were not included in the study. All methods were performed in accordance with the Declaration of Helsinki.

### Statistical analysis

Analyses were performed with the EZR software package (version 3.6.3). Graphs were prepared with GraphPad Prism, version 9. Continuous variables are expressed as the median and interquartile range (IQR) according to their distribution. The data were analyzed with the Shapiro–Wilk test for normality and Brown–Forsythe test for equal variances, followed by the Tukey–Kramer test or Steel–Dwass test for multiple comparisons. Mann–Whitney *U* test was used for comparing two groups, and a Cox proportional-hazards regression model was used for analyzing renal survival in patients with multiple risk factors. Log-rank test was used for comparing renal survival between two groups. A P value of less than 0.05 was considered statistically significant.

## Results

### Histological findings of underlying kidney disorders and patient characteristics

The most prevalent underlying disorders identified by kidney biopsy, the primary endpoint of this study, in the 357 patients were as follows (Table 1): DN (*n* = 100, 28.0%), IgAN (*n* = 99, 27.7%), FGS (*n* = 34, 9.5%), AAV (*n* = 19, 5.3%), MGN (*n* = 15, 4.2%), LN (*n* = 10, 2.8%), AA-Amy (*n* = 9, 2.5%), MPGN (*n* = 9, 2.5%), MNS (*n* = 6, 1.7%), TIN (*n* = 6, 1.7%), Alport (*n* = 5, 1.4%), LC-Amy (*n* = 5, 1.4%), Cryo (*n* = 3, 0.8%), and GBM (*n* = 3, 0.8%). Among the 100 patients with DN, 4 were diagnosed with type 1 DM and 96 with type 2 DM. The patient characteristics in aggregate were as follows: mean (SD) age at kidney biopsy 54.6 (15.9) years old; serum creatinine 3.3 (3.2) mg/dL; eGFR 28.0 (20.1) ml/min/1.73m^2^; proteinuria 2.9 (3.0) g/gCre; hematuria score 1.5 (1.4); HbA1c 6.2 (1.3) %; number of days from kidney biopsy to dialysis 2309 (2359) days; and age at start of dialysis 60.8 (15.3) years old (Table 2).

**Table 1:** Number and percentage of histopathological diagnoses of kidney biopsy by respective dialysis initiation periods

		Period 1 (1986–1999)	Period 2 (2000–2009)	Period 3 (2010–2019)
Total	357	41	160	156
DN	100 (28.0%)	10 (24%)	51 (32%)	39 (25%)
IgAN	99 (27.7%)	15 (37%)	39 (24%)	45 (29%)
FGS	34 (9.5%)	5 (12%)	13 (8%)	16 (10%)
ANCA	19 (5.3%)	2 (5%)	7 (4%)	10 (6%)
Membranous	15 (4.2%)	2 (5%)	9 (6%)	4 (3%)
Lupus	10 (2.8%)	0	6 (4%)	4 (3%)
AA-Amy	9 (2.5%)	0	7 (4%)	2 (1%)
MPGN	9 (2.5%)	2 (5%)	2 (1%)	5 (3%)
MNS	6 (1.7%)	0	2 (1%)	4 (3%)
TIN	6 (1.7%)	1 (2%)	4 (3%)	1 (1%)
Alport	5 (1.4%)	0	3 (2%)	2 (1%)
LC-Amy	5 (1.4%)	0	1 (1%)	4 (3%)
Cryo	3 (0.8%)	0	1 (1%)	2 (1%)
anti-GBM	3 (0.8%)	1 (2%)	1 (1%)	1 (1%)
BNS	1 (0.3%)	0	0	1 (1%)
Others	33 (9.2%)	3 (7%)	14 (9%)	16 (10%)
		ND (*n* = 2), ITG (*n* = 1)	ND (*n* = 4), Minimal (*n* = 2), Mitochon (*n* = 2), Gout (*n* = 2), IgAV (*n* = 1), ING (*n* = 1), CCE (*n* = 1), TMA (*n* = 1)	ND (*n* = 4), ING (*n* = 2), Mitchon (*n* = 1), Gout (*n* = 1), IgAV (*n* = 1), TMA (*n* = 2), ANCA-neg (*n* = 1), UMOD (*n* = 1), OFD-1 (*n* = 1), SRC (*n* = 1), Obesity (*n* = 1)

*DN* diabetic nephropathy, *IgAN* IgA nephropathy, *FGS* focal segmental glomerulosclerosis, *ANCA* antineutrophil cytoplasmic autoantibody-associated nephropathy, *Membranous* membranous glomerulonephritis, *Lupus* lupus nephritis, *Amy AA* amyloid A amyloidosis, *MPGN* membranoproliferative glomerulonephritis, *MNS* malignant nephrosclerosis, *TIN* tubulointerstitial nephritis, *Alport* Alport syndrome, *LC-Amy* light-chain amyloidosis, *Cryo* cryoglobulinemic glomerulonephritis, *anti-GBM* antiglomerular basement membrane antibody-associated glomerulonephritis, *BNS* benign nephrosclerosis, *ND* not diagnosed, *Minimal* minimal change disease, *Mitochon* mitochondria-associated nephropathy, *Gout* gouty nephropathy, *HSP* Henoch–Schonlein purpura, *ING* idiopathic nodular glomerulosclerosis, *Cholesterol* cholesterol thromboembolism, *TMA* thrombomicroangiopathy, *ANCA-neg* antineutrophil cytoplasmic autoantibody-negative crescentic glomerulonephritis, *UMOD* uromodulin-associated kidney disease, *Hemi* status post heminephrectomy, *OFD-1* orofaciodigital syndrome 1 nephropathy, *Sclero* scleroderma nephropathy, *Obesity* obesity-associated nephropathy

**Table 2:** Clinical characteristics of the histopathological kidney diseases

	Number (Male/Female)		Age at renal biopsy	Serum Cre (mg/dL)	eGFR (ml/min/1.73m2)	Proteinurea (g/gCre)	Hematurea score	HbA1c (%)	Days from renal biopsy to dialysis	Age at start of dialysis
DN	100 (73/27)	Median (IQR)	57.5 (49–65)	2.1 (1.5–3.4)	24.2 (14.3–36.5)	3.5 (1.7–7.0)	1.0 (0.0–1.0)	6.6 (6.0–7.8)	971 (380–1928)	62.6 (51.9–69.9)
		Mean (SD)	56.5 (12.0)	3.0 (2.4)	27.0 (16.5)	4.6 (3.8)	1.1 (1.1)	7.0 (1.6)	1441 (1669)	60.4 (12.6)
IgAN	99 (72/27)	Median (IQR)	50 (39–62)	1.7 (1.4–2.5)	30.4 (19.0–42.9)	1.6 (0.7–2.5)	2.0 (1.0–3.0)	5.8 (5.2–6.2)	2743 (1062–4346)	59.7 (46.9–71.5)
		Mean (SD)	49.8 (15.9)	2.9 (3.5)	33.6 (22.3)	1.9 (1.6)	2.1 (1.3)	5.8 (0.9)	3058 (2638)	58.2 (15.8)
FGS	34 (24/10)	Median (IQR)	58.5 (49.8–72.0)	2.0 (1.5–4.2)	26.5 (12.3–34.5)	2.1 (0.7–3.1)	1.0 (0.0–1.0)	5.8 (5.1–6.2)	1739 (596–3728)	65 0 (59.3–73.8)
		Mean (SD)	58.8 (15.7)	3.6 (3.9)	27.0 (19.6)	2.0 (1.3)	0.9 (1.1)	5.7 (0.6)	2335 (2147)	65.2 (13.8)
ANCA	19 (2/17)	Median (IQR)	62.0 (58.0–79.0)	4.0 (2.7–6.2)	9.4 (6.5–17.7)	1.0 (0.4–1.5)	3.0 (1.0–4.0)	5.7 (5.2–6.3)	1086 (0–1773)	73.3 (62.0–81.0)
		Mean (SD)	61.9 (20.0)	5.4 (4.6)	15.3 (16.5)	1.1 (0.8)	2.7 (1.6)	5.8 (0.8)	1560 (2091)	66.2 (18.4)
Membranous	15 (12/3)	Median (IQR)	65.0 (56.0–70.0)	1.5 (1.1–2.6)	40.3 (19.6–58.3)	4.3 (2.4–8.8)	1.0 (0.0–1.0)	6.8 (5.9–7.2)	2321 (1001–4819)	72.2 (64.0–79.7)
		Mean (SD)	63.5 (13.6)	2.3 (2.1)	37.5 (21.1)	5.7 (3.9)	0.9 (1.1)	6.7 (1.1)	2943 (2301)	71.5 (12.7)
Lupus	10 (3/7)	Median (IQR)	45.0 (29.0–57.0)	1.8 (1.1–3.9)	31.4 (10.5–51.2)	2.0 (0.4–3.6)	1.0 (0.0–2.3)	4.7 (4.4–5.5)	1361 (29–3241)	47.3 (40.4–60.5)
		Mean (SD)	43.2 (17.8)	2.2 (1.4)	36.5 (27.1)	2.1 (1.8)	1.2 (1.4)	4.8 (0.8)	2307 (3126)	49.5 (13.2)
Amy AA	9 (1/8)	Median (IQR)	64.0 (61.0–68.5)	2.6 (2.0–3.0)	16.8 (13.4–20.3)	1.5 (0.5–3.4)	2.0 (1.0–3.0)	5.6 (5.0–5.9)	1051 (430–1967)	68.0 (62.1–72.3)
		Mean (SD)	62.9 (8.8)	3.0 (1.8)	17.0 (6.5)	1.9 (1.6)	2.0 (1.1)	5.4 (0.5)	1131 (763)	66.0 (8.3)
MPGN	9 (7/2)	Median (IQR)	41.0 (35.0–69.0)	1.7 (1.4–5.2)	33.6 (13.7–42.8)	1.3 (0.6–5.3)	0.5 (0.0–1.0)	5.5 (5.1–5.7)	5118 (2522–5907)	58.6 (46.9–82.4)
		Mean (SD)	49.8 (22.6)	3.0 (2.8)	29.3 (15.8)	2.4 (2.6)	0.5 (0.6)	5.4 (0.3)	4892 (2911)	63.2 (20.7)
MNS	6 (6/0)	Median (IQR)	55.0 (39.3–61.3)	6.0 (2.5–9.2)	8.6 (6.0–28.7)	0.4 (0.1–1.5)	1.0 (0.0–2.0)	5.1 (4.6–5.6)	1676 (185–3873)	58.8 (54.0–63.2)
		Mean (SD)	51.7 (11.0)	6.1 (4.0)	16.6 (17.6)	0.9 (1.3)	1.0 (1.2)	5.1 (0.7)	2332 (2872)	58.1 (6.2)
TIN	6 (1/5)	Median (IQR)	55.0 (47.8–61.8)	3.6 (2.8–5.2)	11.1 (8.9–16.5)	0.2 (0.1–0.6)	0.0 (0.0–1.0)	5.4 (4.9–5.9)	2605 (1952–5409)	60.3 (54.1–77.4)
		Mean (SD)	55.0 (9.6)	4.3 (2.6)	12.0 (5.3)	0.3 (0.3)	0.3 (0.5)	5.4 (0.6)	3301 (1831)	64.1 (13.3)
Alport	5 (4/1)	Median (IQR)	38.0 (27.5–63.5)	1.5 (1.3–3.0)	47.3 (15.9–55.0)	0.7 (0.3–10.7)	1.0 (0.0–3.0)	5.7 (5.5–6.4)	3066 (2616–5276)	53.2 (36.3–73.0)
		Mean (SD)	44.0 (23.1)	2.0 (1.0)	37.8 (20.3)	4.5 (5.9)	1.4 (1.7)	5.9 (0.4)	3770 (1967)	54.3 (22.0)
Amy AL	5 (4/1)	Median (IQR)	58.0 (48.5–68.0)	1.0 (0.8–2.9)	60.5 (23.4–67.1)	5.6 (3.7–7.9)	0.0 (0.0–0.0)	5.1 (4.9–6.8)	1412 (708–2939)	58.8 (53.2–74.9)
		Mean (SD)	58.2 (10.0)	1.7 (1.4)	48.3 (24.2)	5.8 (2.5)	0.0 (0.0)	5.7 (1.1)	1741 (1254)	63.0 (11.9)
Cryo	3 (3/0)	Median (IQR)	55.0 (46.0–75.0)	1.5 (0.8–2.4)	36.9 (24.8–78.4)	3.0 (2.9–3.2)	1.0 (1.0–4.0)	5.3 (5.2–5.7)	1122 (0–4341)	66.9 (49.1–75.0)
		Mean (SD)	58.7 (14.8)	1.6 (0.8)	46.7 (28.1)	3.0 (0.2)	2.0 (1.7)	5.4 (0.2)	1821 (2253)	63.7 (13.3)
GBM	3 (1/2)	Median (IQR)	76.0 (54.0–81.0)	8.6 (7.5–8.9)	4.5 (4.3–5.1)	0.2 (0.2–0.6)	4.0 (4.0–4.0)	5.9 (5.2–6.2)	3 (1–8)	76.0 (54.0–81.0)
		Mean (SD)	70.3 (14.4)	8.3 (0.7)	4.6 (0.4)	0.4 (0.2)	4.0 (0.0)	5.7 (0.5)	4 (4)	70.3 (14.4)
Total	357	Median (IQR)	56.0 (44.0–66.0)	2.0 (1.5–3.7)	24.3 (12.6–39.2)	1.9 (0.7–3.7)	1.0 (0.0–3.0)	6.0 (5.4–6.6)	1586 (455–3196)	62.0 (50.7–71.9)
	(231/126)	Mean (SD)	54.6 (15.9)	3.3 (3.2)	28.0 (20.1)	2.9 (3.0)	1.5 (1.4)	6.2 (1.3)	2309 (2359)	60.8 (15.3)

Hematuria grade: 0, < 1 red blood cell (RBC) per high-power field (HPF); 1, 1 to 5 RBCs per HPF; 2, 6 to 10 RBCs per HPF; 3, 11 to 30 RBCs per HPF; and 4, > 30 RBCs per HPF. The data are shown as median and interquartile range (IQR) and mean and standard deviation (SD)

The mean age at the time of kidney biopsy could be categorized as follows: 40 to 49 years for IgAN, LN, MPGN and Alport; 50 to 59 years for DN, FGS, MNS, TIN, LC-Amy, and Cryo; 60 to 69 years for AAV, MGN, and AA-Amy; and 70 to 79 years for anti-GBM. Renal function at the time of kidney biopsy was as follows: a mean eGFR of less than 30 mL/min/1.73m^2^ was observed in DN, FGS, AAV, AA-Amy, MPGN, MNS, TIN, and anti-GBM; and a median eGFR over 30 mL/min/1.73m^2^ in IgAN, LN, MGN, Alport, LC-Amy, and Cryo. The mean proteinuria values at the time of the kidney biopsy were as follows: less than 1 g/gCre was observed in MNS, TIN, Alport, and anti-GBM; 1 g to 3 g in IgAN, FGS, AAV, LN, AA-Amy, and MPGN; and over 3 g in DN, MGN, LC-Amy, and Cryo. A mean hematuria grade above 2.0 at the time of kidney biopsy was observed in IgAN, AAV, AA-Amy, and anti-GBM. The mean duration from kidney biopsy to dialysis initiation exceeded 2000 days in IgAN, FGS, MGN, LN, MPGN, MNS, TIN, and Alport (Table 2).

### Underlying kidney diseases across the three periods: a subgroup analysis

A subgroup analysis examining the prevalence of underlying diseases across the three periods (1985 to 1999; 2000 to 2009; and 2010 to 2019) revealed that DN and IgAN were the two predominant underlying etiologies for dialysis initiation in each period (Table 1). The frequency distribution of kidney diseases remained consistent throughout the three periods.

### Underlying kidney diseases in each age group at dialysis initiation: a subgroup analysis

At dialysis initiation, the predominant underlying diseases in each of the age groups were as follows (Table 3): patients aged 30 to 39 years, IgAN and DN; patients aged 40 to 49 years, IgAN, DN, LN and AAV; patients aged 50 to 59 years, DN, IgAN, FGS, LC-Amy, MNS, and MPGN; patients aged 60 to 69 years, DN, IgAN, FGS, AAV, AA-Amy and MGN; patients aged 70 to 79years, DN, IgAN, FGS, MGN, and AAV; and patients aged 80 years and older, IgAN, AAV and FGS. DN and IgAN were the two primary underlying causes of dialysis initiation in all age groups. IgAN was more prevalent than DN in patients aged < 50 years, whereas DN was more prevalent than IgAN in patients aged > 50 years and < 80 years. FGS has emerged as the third most prevalent underlying disease in patients over 50 years of age, while AAV manifests as an underlying disease in patients aged > 60.

**Table 3:** Number of patients with the respective histopathology categorized by age group at dialysis initiation

Age at induction of dialysis	< 20	20 = < age < 30	30 = < age < 40	40 = < age < 50	50 = < age < 60	60 = < age < 70	70 = < age < 80	80 = < age
Number of cases	3	8	24	51	65	96	79	31
	IgAN (*n* = 1)	IgAN (*n* = 4)	IgAN (*n* = 10)	IgAN (*n* = 16)	DN (*n* = 21)	DN (*n* = 33)	DN (*n* = 22)	IgAN (*n* = 6)
	ANCA (*n* = 1)	DN (*n* = 1)	DN (*n* = 6)	DN (*n* = 14)	IgAN (*n* = 19)	IgAN (*n* = 22)	IgAN (*n* = 21)	ANCA (n = 5)
	Mitchon (*n* = 1)	Lupus (*n* = 1)	FGS (*n* = 2)	Lupus (*n* = 4)	FGS (*n* = 5)	FGS (*n* = 12)	FGS (*n* = 8)	FGS (*n* = 5)
		Alport (*n* = 1)	Mitchon (*n* = 2)	ANCA (*n* = 3)	LC Amy (*n* = 3)	ANCA (*n* = 5)	MGN (*n* = 6)	DN (*n* = 3)
		UMOD (*n* = 1)	Lupus (*n* = 1)	FGS (*n* = 2)	MNS (*n* = 3)	AA Amy (*n* = 5)	ANCA (*n* = 5)	MGN (*n* = 3)
			MPGN (*n* = 1)	Not Dx (*n* = 2)	MPGN (*n* = 3)	MGN (*n* = 4)	AA Amy (*n* = 3)	MPGN (*n* = 2)
			OFD-1 (*n* = 1)	Alport (*n* = 1)	Alport (*n* = 2)	Not Dx (*n* = 3)	MPGN (*n* = 2)	LC Amy (*n* = 1)
			HSP (*n* = 1)	AA Amy (*n* = 1)	TIN (*n* = 2)	Lupus (*n* = 2)	Not Dx (*n* = 2)	Alport (*n* = 1)
				Cryo (*n* = 1)	Not Dx (*n* = 2)	MNS (*n* = 2)	TIN (*n* = 1)	anti-GBM (*n* = 1)
				Gout (*n* = 1)	Obesity (*n* = 1)	Gout (*n* = 1)	Lupus (*n* = 1)	ING (*n* = 1)
				ING (*n* = 1)	anti-GBM (*n* = 1)	LC Amy (*n* = 1)	ING (*n* = 1)	Not Dx (*n* = 1)
				MGN (*n* = 1)	HSP (*n* = 1)	TIN (*n* = 1)	Gout (*n* = 1)	TIN (*n* = 1)
				MNS (n = 1)	Lupus (*n* * n* = 1)	Cryo (*n* = 1)	anti-GBM (* n* = 1)	TMA (*n* = 1)
				MPGN (*n* = 1)	MGN (*n* = 1)	BNS (*n* = 1)	ANCA-neg GN (*n* = 1)	
				Sclero (*n* = 1)		ITG (n = 1)	Cholesterol (*n* = 1)	
				TIN (*n* = 1)		Minimal (*n* = 1)	Cryo (*n* = 1)	
						TMA (*n* = 1)	Minimal (*n* = 1)	
							TMA (*n* = 1)	

### Clinical characteristics of patients with DN among the three periods: a subgroup analysis

**Fig. 1:**
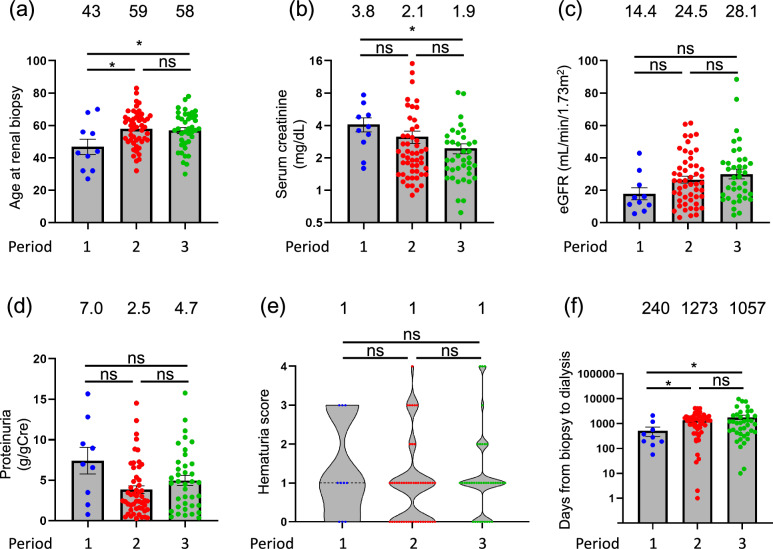
Comparison of clinical characteristics of patients with diabetic nephropathy at biopsy according to 3 periods of dialysis initiation (1985 to 1999, 2000 to 2009, and 2010 to 2019). (**a**) Age at kidney biopsy. Age was significantly lower in period 1 than in period 2. (**b**) Age at dialysis initiation. Age was significantly lower in period 1 than in periods 2 and 3. (**c**) Estimated glomerular filtration rate (eGFR) at kidney biopsy. eGFR was significantly lower in period 1 than in periods 2 and 3. (**d**) Proteinuria at kidney biopsy. Proteinuria was significantly lower in period 1 than in period 2. (**e**) Serum creatinine at kidney biopsy. Serum creatinine was significantly lower in period 1 than in periods 2 and 3. (**f**) Number of days between kidney biopsy and dialysis initiation. The number of days was significantly lower in period 1 than in periods 2 and 3. Median for each group is shown above each bar. *p < 0.05; *ns* not significant

At dialysis initiation, in periods 1, 2, and 3 patients with DN had a median age of 43, 59, and 58 years, respectively; median serum Cre of 3.8, 2.1, and 1.9 mg/dL; median eGFR of 14.4, 24.5, and 28.1 mL/min/1.73m^2^; median proteinuria of 7.0, 2.5, and 4.7 g/gCre; median hematuria grade of 1, 1, and 1; and median number of days between kidney biopsy and dialysis of 240, 1273, and 1057. Patients in period 1 were significantly younger than those in periods 2 and 3, their renal function was significantly impaired, and the time to dialysis was significantly shorter (Fig. 1).

### Clinical characteristics of patients with IgAN among the three periods: a subgroup analysis

**Fig. 2:**
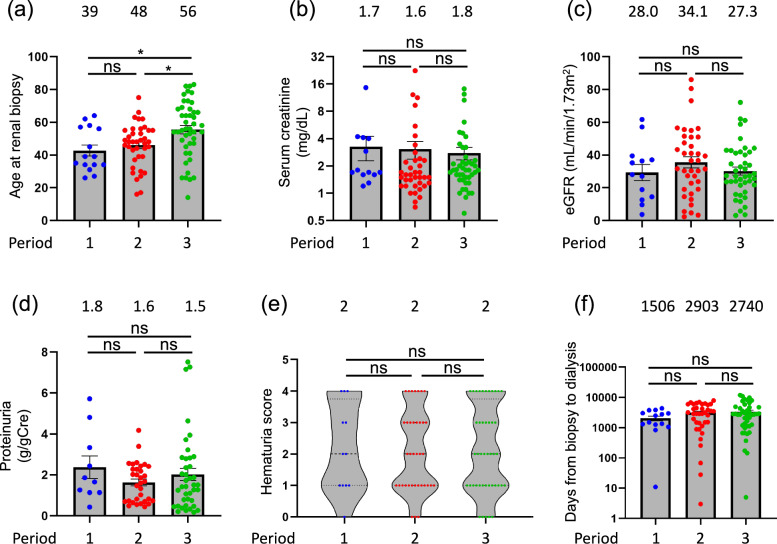
Comparison of clinical characteristics of patients with immunoglobulin A nephropathy at kidney biopsy according to 3 periods of dialysis initiation (1985 to 1999, 2000 to 2009, and 2010 to 2019). (**a**) Age at kidney biopsy. Age was significantly lower in periods 1 and 2 than in period 3. (**b**) Age at dialysis initiation. Age was significantly lower in periods 1 and 2 than in period 3. (**c**) Estimated glomerular filtration rate (eGFR) at kidney biopsy. eGFR was similar in the 3 periods. (**d**) Proteinuria at kidney biopsy. Proteinuria was similar in the 3 periods. (**e**) Serum creatinine at kidney biopsy. Serum creatinine was similar in the 3 periods. (**f**) Number of days between kidney biopsy and dialysis initiation. The number of days was similar in the 3 periods. Median for each group is shown above each bar. *p < 0.05; *ns* not significant

At dialysis initiation, in periods 1, 2, and 3 patients with IgAN exhibited median ages of 39, 48, and 56 years, respectively; median serum Cre levels of 1.7, 1.6, and 1.8 mg/dL; median eGFR of 28.0, 34.1, and 27.3 mL/min/1.73m^2^; median proteinuria levels of 1.8, 1.6, and 1.5 g/gCre; median hematuria grade of 2, 2; and 2; and median intervals between kidney biopsy and dialysis of 1506, 2903, and 2740 days. The age at the time of kidney biopsy was significantly higher in period 3 than in period 1 and 2, however, renal function and time to dialysis were comparable (Fig. 2).

### Risk factors associated with reduced time to dialysis initiation: the secondary endpoint

The analysis of 14 variables in the whole cohort (*n* = 357) using a Cox regression model identified decreased eGFR, elevated proteinuria, advanced age, and histologically confirmed DN as independent risk factors for a shorter time to dialysis initiation (Table 4).

**Table 4:** Coefficients of multivariate Cox regression analysis of dialysis initiation in all 357 cases

Variable	Hazard ratio	Lower 95% CI	Upper 95% CI	p value
eGFR, 1 ml/min/1.73m^2^	0.968	0.959	0.976	< 0.0001
UP, 1 g/gCre	1.112	1.064	1.163	< 0.0001
Age, 1 year	1.014	1.004	1.024	0.005
DN, yes	1.655	1.140	2.403	0.008
LN, yes	2.178	0.996	4.765	0.051
AA-Amy, yes	1.762	0.838	3.704	0.135
MGN, yes	0.595	0.285	1.242	0.167
MPGN, yes	0.476	0.144	1.567	0.222
UB, 1 score	0.952	0.867	1.046	0.304
Sex, 1 for male	1.134	0.864	1.490	0.365
IgAN, yes	1.194	0.813	1.754	0.365
FGS, yes	1.195	0.756	1.891	0.446
MNS, yes	0.722	0.250	2.083	0.547
AAV, yes	1.146	0.609	2.157	0.672

*CI* confidence interval

### Comparison between DN and IgAN: a subgroup analysis

**Fig. 3:**
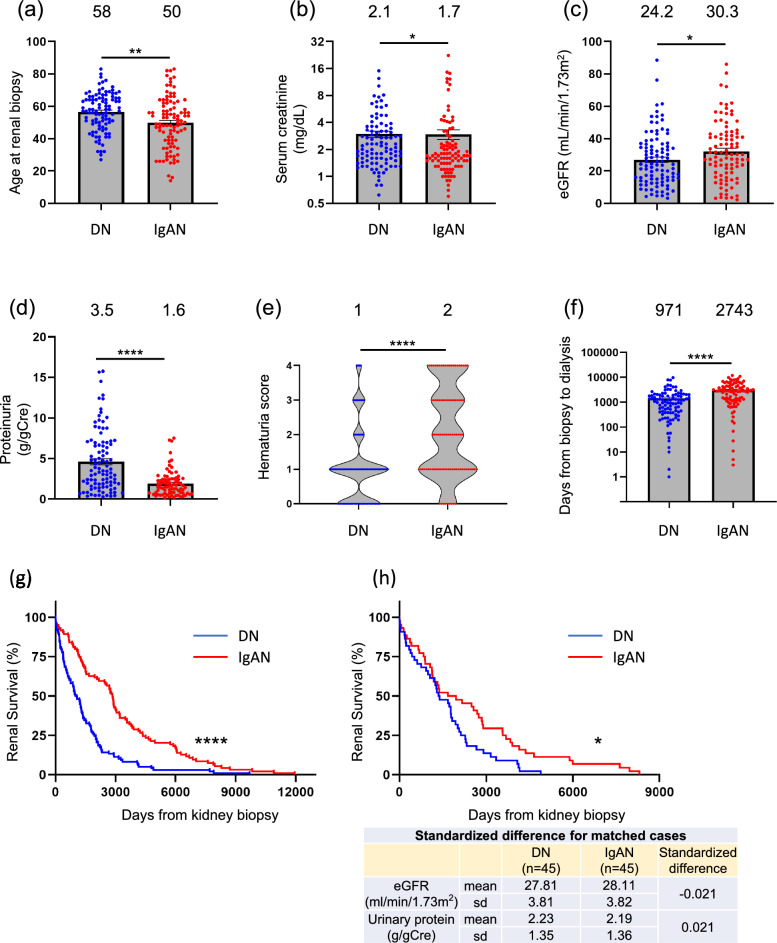
Comparison of clinical characteristics of patients with diabetic nephropathy and immunoglobulin A nephropathy in the overall time periods. Age at kidney biopsy (**a**), serum creatinine (**b**) and proteinuria (**d**) were significantly greater in DN group than in IgA group, whereas eGFR (**c**), hematuria score, and days from biopsy to dialysis (**f**) was significantly smaller. (**g**) Overall renal survival was significantly better in IgAN group when compared with DN group. (**h**) In groups with propensity score matched for eGFR and proteinuria showed significantly better overall renal survival in IgAN group when compared with DN group. Standardized difference between the matched groups for both eGFR and proteinuria were low enough so that the two groups were fair to compare. Median for each group is shown above each bar. *p < 0.05; **p < 0.01; ****p < 0.0001; *ns* not significant

When compared with the IgAN group, the DN group exhibited a higher age at the time of kidney biopsy, elevated serum Cre and proteinuria levels, reduced eGFR and hematuria grade, and a shorter interval between kidney biopsy and dialysis. Renal survival was significantly lower in the DN group than in the IgAN group. Furthermore, Cox regression analysis of time to dialysis initiation for 199 patients with DN or IgAN revealed that eGFR, proteinuria, and DN, but not age, were independent risk factors for a shorter time to dialysis (Table 5). Even when both groups were matched by the propensity score for eGFR and proteinuria, renal survival was significantly shorter in patients with DN than in those with IgAN (Fig. 3). The validity of the matched groups was confirmed by calculating standardized differences, which were approximated to zero.

**Table 5:** Coefficient of multivariate analysis by cox regression analysis of dialysis initiation for 199 cases (DN and IgAN)

Variable	Hazard ratio	Lower 95% CI	Upper 95% CI	p value
eGFR, 1 ml/min/1.73m2	0.962	0.950	0.974	< 0.0001
UP, 1 g/gCre	1.143	1.083	1.206	< 0.0001
DN, yes	1.462	1.018	2.099	0.0398
Age, 1 year	0.995	0.981	1.009	0.502
UB, 1 score	0.964	0.851	1.091	0.557
Sex, 1 for male	1.073	0.756	1.523	0.692

*CI* confidence interval

### Underlying kidney disease in other dialysis patients

Of the patients who did not undergo kidney biopsy, 1025 patients who initiated dialysis were identified from the medical records at the time of dialysis initiation. (1) ADPKD or polycystic kidney disease can be diagnosed based on the family history and imaging studies alone (*n* = 320, 31.2%). The ratio of kidney biopsy was 0%. (2) DN can also be diagnosed based on a history of DM, significant proteinuria, and diabetes-specific complications, such as retinopathy and foot gangrene (*n* = 285, 27.8%). The percentage of kidney biopsies performed was 26.0% (100 of 385). (3) Primary glomerulonephritis such as IgAN or FGS can be considered in patients with a long-standing history of proteinuria and hematuria at a young age. However, when the patient had a brief history of diabetes mellitus and no diabetes-specific complications, primary glomerular diseases other than diabetes may have been the direct cause of renal failure (*n* = 220, 21.4%). The percentage of kidney biopsies performed was 53.9% (257 of 477). (4) BNS, arteriosclerosis, or nephrosclerosis, the most commonly used term for kidney disease in Japan, is applied to elderly patients with systemic atherosclerosis, including hypertension, heart disease, low proteinuria, and small kidney size. In such cases, rather than considering the cause of renal failure to be the progression of arteriosclerosis, it was hypothesized that concurrent heart disease, liver disease, malignancy, and its treatment may contribute to the progression of renal failure (*n* = 48, 4.7%). The percentage of kidney biopsies performed was 2.0% (1 of 49).

### Comprehensive characterization of patients with IgAN, including clinical and histological grades at kidney biopsy

**Fig. 4:**
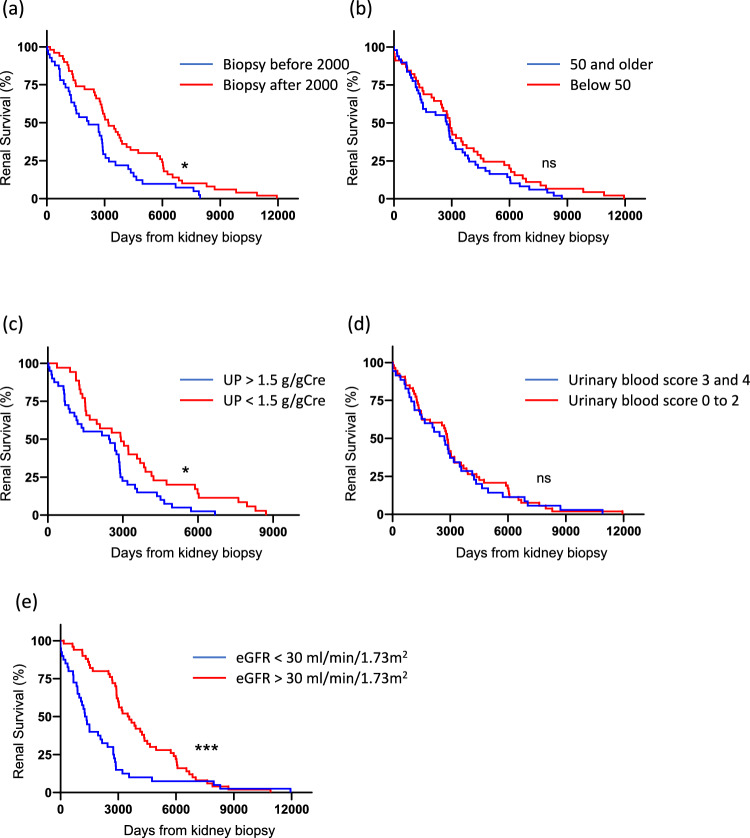
Renal survival in subgroups of patients with IgAN (**a**) Renal survival in IgAN patients diagnosed by kidney biopsy prior to 2000 was statistically superior to that of patients diagnosed after 2000. (**b**) No statistically significant difference in renal survival was observed between IgAN patients aged ≥ 50 years and those aged < 50 years at the time of kidney biopsy. (**c**) Renal survival in IgAN patients with lower proteinuria (< 1.5 g/gCre) was superior to those with higher proteinuria (> 1.5 g/gCre) at the time of kidney biopsy. (**d**) No statistically significant difference in renal survival was observed between IgAN patients in the high-grade urinary blood group and the low-grade group. (**e**) Renal survival in IgAN patients with higher eGFR (> 30 ml/min/1.73 m^2^) was superior to those with lower eGFR (> 30 ml/min/1.73 m^2^) at the time of kidney biopsy. *p<0.05; ***p<0.001; ns not significant

The clinical and histological grade is shown in Table 6. The prevalence of segmental sclerosis is as high as 77.9%. Eighty percent of patients were classified as having clinical grade III disease (Table 6a). Although there were some missing data, no patients were observed in the combinations of clinical grade I and histological grade I or clinical grade II and histological grades II, III, and IV (Table 6b). We stratified IgAN patients into two groups based on the biopsy year (before or after 2000) (Fig. 4a), age (Fig. 4b), proteinuria (Fig. 4c), urinary blood level (Fig. 4d), and eGFR (Fig. 4e). The median of each factor was chosen to divide the patients into two groups. The group of patients biopsied after 2000 (*p* = 0.0128), those with lower-level proteinuria (*p* = 0.0101), and those with more preserved eGFR (*p* = 0.0007) demonstrated statistically better renal survival than their respective counterparts. Moreover, multivariate Cox regression analysis of 99 IgAN patients revealed that renal function (serum creatinine) and proteinuria were independent risk factors for early dialysis initiation in both Model 1 and Model 2 (Table 7).

**Table 6:** Histological and clinical grade in IgAN patients. a) Number and percentage of MEST-C score, Histological and Clinical grade in IgAN patients. b) Classification according to respective Histological and clinical grades in IgAN patients

a)		number	percent
M	score 0	76	88.4%
	score 1	10	11.6%
E	score 0	82	95.3%
	score 1	4	4.7%
S	score 0	19	22.1%
	score 1	67	77.9%
T	score 0	41	47.7%
	score 1	15	17.4%
	score 2	30	34.9%
C	score 0	71	83.5%
	score 1	14	16.5%
Histological grade	Grade I	12	14.5%
	Grade II	20	24.1%
	Grade III	26	31.3%
	Grade IV	25	30.1%
	AC	17	20.5%
	C	66	79.5%
	Grade I AC	1	1.2%
	Grade I C	11	13.3%
	Grade II AC	4	4.8%
	Grade II C	16	19.3%
	Grade III AC	4	4.8%
	Grade III C	22	26.5%
	Grade IV AC	7	8.4%
	Grade IV C	18	21.7%
Clinical grade	Grade I	12	15%
	Grade II	4	5%
	Grade III	66	80%

b)		Histological grade			
		I	II	III + IV	
Clinical grade	I	0	2 (3.3%)	8 (13.3%)	10 (16.7%)
	II	3 (5%)	0	0	3 (5%)
	III	5 (8.3%)	9 (15%)	33 (55%)	47 (78.7%)
		8 (13.3%)	11 (18.3%)	41 (68.3%)	

*M* mesantial score, *E* endothelial score, *S* segmental score, *T* tubule atrophy score, *C* crescent score, *A* acute lesion, *C* chronic lesion

**Table 7:** Coefficients of multivariate analysis by cox regression analysis of dialysis initiation for 99 IgAN cases

Model 1	Hazard ratio	Lower 95% CI	Upper 95% CI	P value
Age, 1 year	1.012	0.9931	1.031	0.227
Cre, 1 mg/dL	1.289	1.195	1.400	< 0.0001
UP, 1 g/gCre	1.161	1.003	1.332	0.0379
Urinary blood, 1 score	1.053	0.8764	1.259	0.5775
Sex, male	0.8793	0.5298	1.509	0.628
Model 2	Hazard ratio	Lower 95% CI	Upper 95% CI	P value
Biopsy after 2000, yes	1.173	0.7328	1.888	0.5063
Age, 1 year	1.011	0.9921	1.031	0.2663
Cre, 1 mg/dL	1.285	1.19	1.396	< 0.0001
UP, 1 g/gCre	1.154	0.9948	1.327	0.0494
Urinary blood, 1 score	1.062	0.8825	1.274	0.5179
Sex, male	0.8671	0.5214	1.49	0.5926

*CI* confidence interval

### The prevalence of cigarette smoking and hypertension

Among the patients who were initiated on dialysis, the smoking prevalence was 337 out of 1025 (35.3%) for those who had not undergone a kidney biopsy and 166 out of 357 (47.5%) for those who had undergone a kidney biopsy. Regarding hypertension, in the case of kidney biopsy, 339 of 357 patients (96%) were taking antihypertensive medication. Conversely, in the case of nonkidney biopsy, 960 of 1025 patients (97.1%) were taking antihypertensive medication.

## Discussion

This retrospective cohort study elucidated histologically diagnosed kidney diseases responsible for ESKD. Tables 1, 2, and 3 describe the clinical characteristics of the study cohort. Lower eGFR, higher proteinuria, DN, and advanced age were significant risk factors for early dialysis initiation (Table 4). DN and IgAN are the two primary causes of ESKD. We analyzed the clinical characteristics of DN and IgAN and compared these two diseases at the time of kidney biopsy, as illustrated in Figs. 1, 2, and 3.

Although the number of patients on dialysis has been reported to be increasing in many countries, few studies have evaluated the underlying diseases, as confirmed by kidney biopsy, that led to dialysis initiation [1–3]. In patients with DM, accurate diagnosis of kidney histology is a crucial indicator of the future course of treatment when the kidneys are affected. Diagnosing DN using classification schemes facilitates improved communication between renal pathologists and clinicians, provides a logistical structure for prognostic and interventional studies, and enhances clinical management and efficiency. In accordance with this approach, Tervaert et al. proposed a pathologic classification of DN [6]. Furthermore, the Committee on Practical Guidance for Kidney Biopsy in Japan published a second edition of its kidney biopsy guidebook, suggesting that in patients with DM and renal complications, a kidney biopsy can provide a wide range of information, including: (1) presence of typical DN, (2) existence of arteriolar hyalinosis without typical DN, (3) presence of glomerular lesions other than DN, and (4) a mixture of these lesions [7]. Even in patients with clinical information suggesting typical DM accompanied by nephrotic-range proteinuria, retinopathy, and neuropathy, kidney biopsy provides pathological information that can inform treatment strategies to improve renal prognosis. Additional studies on kidney biopsy in DM patients support this approach [8, 9].

Previous reports have indicated that DN, chronic glomerulonephritis, and hypertension-related nephropathy are the three primary causes of dialysis initiation in Japan [1, 2] based on questionnaire surveys. The current study demonstrated that IgAN, FGS, MGN, and MPGN are types of chronic glomerulonephritis leading to dialysis. The pathogenesis of hypertension-associated nephropathy as an initiating factor in chronic kidney disease remains unclear [10, 11]. Moreover, the data presented herein suggest that BNS is not a major direct cause of ESKD in patients requiring kidney biopsy, a finding that is consistent with the study by Sumida et al., who investigated the renal prognosis of patients diagnosed with BNS by kidney biopsy [12]. In support of this, a meta-analysis examining kidney dysfunction in ten trials with 114,000 person-years in patients with nonmalignant or benign hypertension revealed that patients treated for hypertension did not exhibit a lower risk of renal dysfunction [13]. Furthermore, after 8–12 years of follow-up, intensive blood pressure control in patients with hypertension was not superior to normal blood pressure control with regard to renal function [14]. However, patients in the intensive blood pressure control group with proteinuria exceeding 0.22 g/gCre demonstrated a potential benefit of preserved renal function [14], suggesting that the underlying kidney condition, such as chronic glomerulonephritis or glomerulopathy accompanied by proteinuria, is susceptible to hypertension resulting in declined kidney function. Therefore, a combination of hypertension, chronic glomerulonephritis, and proteinuria may be a significant contributor to progressively declining kidney function, ultimately leading to dialysis initiation. As most kidney biopsies in older adults are associated with hypertension, the involvement of hypertension in the underlying kidney disease should be carefully evaluated. Indeed, a previous article reported that kidney biopsy of hypertensive patients revealed that 16% of the patients had primary nephritis, 19% FGS, 43% MNS, and 22% BNS [15]. This suggests that approximately 80% of hypertensive patients have glomerulopathy or glomerulonephritis, a potential factor for poor kidney prognosis, which are histologically distinctive from BNS.

Conversely, others studies have reported that biopsy-proven BNS was associated with poor renal prognosis, although in this particular study, 71% of participants had proteinuria with a maximum proteinuria of 5.1 g/day [16], suggesting that an underlying glomerulopathy could be present in some patients in addition to BNS. As delineated in the methodology section, BNS should present with intact glomeruli, and arterioles or arteries being the primary site of injury. Therefore, by definition, BNS does not typically manifest as proteinuria. This analysis corroborates our finding that BNS is an infrequent direct cause of ESKD in patients requiring kidney biopsy.

Although FGS in young patients has garnered significant attention regarding genetic abnormalities, its occurrence in clinical practice is infrequent, with majority FGS patients being over 50 years of age. Determining whether FGS is secondary to hypertension, or a primary disease, presents a diagnostic challenge. Similarly, ascertaining whether hypertension is secondary to FGS is equally problematic. It is well-established that FGS can develop due to numerous underlying conditions [17], thus rendering it a heterogeneous diagnosis in terms of etiology. The Columbia classification for FGS [18] provides a useful framework; however, in practice, patients often do not meet the specified criteria, resulting in a diagnosis of “not otherwise specified*”*. Future research is necessary to address this diagnostic limitation.

Several additional considerations merit further discussion in this study: the underlying kidney diseases in other dialysis patients; a more detailed characterization of IgAN patients, including clinical and histological grades at kidney biopsy; the prevalence of cigarette smoking and hypertension, both of which are factors associated with ESKD; a comparison between this study and the Japan Renal Biopsy Registry (J-RBR); a comparison between this study and previously reported [12] BNS prognoses; the potential association between FGS and BNS; complications related to kidney biopsy under the indication criteria in this study; the consistency of indication criteria for kidney biopsy throughout the study period; and the prevalence of BNS among all kidney biopsies, including patients without ESKD.

The underlying kidney diseases in other dialysis patients were also analyzed as described in the results section. ADPKD was the most prevalent renal disease, because our institution specializes in its treatment. Consequently, the distribution of diseases, excluding ADPKD, may be comparable to that of a general institution.

A more comprehensive characterization of patients with IgAN, including clinical and histological grades at kidney biopsy, was conducted as described in Table 6. Furthermore, as illustrated in Fig. 2, the time required to reach ESKD after biopsy tended to be longer in period 3 than in period 1. Since 2000, tonsillectomy and glucocorticoid pulse therapy have been implemented for IgAN at our institution, resulting in a significant improvement in the prognosis of IgAN. Notably, all 16 patients with creatinine levels exceeding 2.0 mg/dL ultimately required dialysis; however, the interval to dialysis initiation was prolonged. Nevertheless, some patients did not receive this treatment because of their advanced age. Although Fig. 4 demonstrated that patients with biopsies after 2000 obtained statistically better survival than their counterparts, Table 7 described only renal function and proteinuria were independent risk factors for early dialysis initiation. This may be due to the fact that patients with biopsies after 2000 presented with worse renal function (median serum creatinine 2.1 mg/dL vs 1.6 mg/dL) and higher-level proteinuria (median proteinuria 1.8 g/gCre vs 1.3 g/gCre).

The prevalence of cigarette smoking and hypertension, both of which are associated with ESKD, is of significant importance. As demonstrated in the results section, the prevalence of smoking was as high as 35.3% for patients who initiated dialysis. Considering that the prevalence of smoking in Japan is 16.7%, this elevated rate may be a contributing factor to the progression of renal failure. Regarding hypertension, 97% of patients who initiated dialysis were taking antihypertensive medication as demonstrated in the results section. This observation suggests that hypertension is associated with progression to ESKD, irrespective of the underlying disease. This finding indicates that treatment modalities targeting the underlying disease in addition to antihypertensive agents may be necessary.

A comparative analysis between this study and the Japan Renal Biopsy Registry (J-RBR) warrants further consideration. J-RBR compiled data on renal biopsies conducted in Japan. The data revealed that IgAN, DN, FGS, MCD, MGN, and other conditions were prevalent across all age groups, whereas AAV and BNS showed an increased incidence in elderly patients. BNS is observed in a significant proportion of elderly patients who present with low proteinuria and preserved renal function. A recent classification of nephrosclerosis into malignant nephrosclerosis and essential hypertension/arteriosclerosis was implemented for data collection. However, there is a dearth of reports on the renal prognosis for each specific disease entity, which remains an area for future investigation within the J-RBR [19–22].

Regarding a comparison between this study and previously reported [12] BNS prognoses, in 2016, Sumida et al. conducted a study of cases in which the pathologist's report described nephrosclerosis [12]. In this study, the diagnosis was made without EM in cases with predominantly LM and negative IgG and IgA test results. Subsequently, in the present study, kidney biopsy specimens were re-examined by multiple pathologists. In some instances, where the initial diagnosis of nephrosclerosis was based on a high number of global sclerosing glomeruli and a high degree of atherosclerosis, one or more focal segmental sclerosing glomeruli were newly identified. The diagnosis of FGS was reassessed in cases of partial IgM deposition by IF and foot process effacement by EM, and these cases were excluded from the BNS. Additionally, genetic testing was performed in patients with a family history of renal disease, and those diagnosed with Alport or UMOD were excluded from the BNS. Furthermore, patients with onion skin lesions were reclassified as having MNS or Sclero and excluded from BNS based on clinical features, including hyperreninemia and rapid deterioration of renal function. Because of these iterative procedures, the number of nephrosclerosis cases with a final diagnosis of arteriosclerosis (BNS) alone was reduced from the list of diseases causing dialysis induction to one. Conversely, renal prognosis of patients with BNS and reduced renal function who discontinued hospital visits was not observed. Patients who succumbed to extrarenal lesions during the course of the disease were lost to follow-up.

In addition to the aforementioned discussion concerning FGS, the potential association between FGS and BNS warrants further examination. The 34 cases diagnosed with FGS in this study excluded primary FGS, in which the primary disease, such as a hereditary condition that causes FGS, was evident. Consequently, further classification of FGS is not feasible. However, most cases diagnosed with FGS exhibit multilayered fibroelastosis of the renal arteries and are diagnosed as coexisting arteriosclerosis and arteriolosclerosis, suggesting a significant association between FGS and renal atherosclerosis. Furthermore, many patients with ESKD present with high-grade proteinuria, a characteristic that differs from BNS by definition because glomerulopathy does not occur in BNS. This observation may indicate the susceptibility of BNS patients to progress to secondary FGS.

Complications associated with kidney biopsy under the specified indications can be delineated as follows: A Silverman needle was utilized between 1985 and 1997. Subsequently, an automatic biopsy needle is used. During the former period, renal hemorrhage following kidney biopsy was prevalent; however, only one patient underwent endovascular treatment, and no patient required surgical nephrectomy. In the latter period, the incidence of renal hemorrhage after kidney biopsy decreased; nevertheless, endovascular treatment was performed in two patients. The guidelines established by the Japanese Society of Nephrology [7] stipulate that kidney biopsy in patients at high risk of hemorrhage should be conducted with informed consent within the same institution, and that renal biopsy should be performed in an institution where hemostatic treatment following renal bleeding is feasible [23] and when the patient has been provided with comprehensive explanation and consent. This protocol was approved by our hospital.

Consistent indication criteria for kidney biopsy throughout the study period was a critical consideration. This investigation demonstrated that serum creatinine levels at the time of kidney biopsy in diabetic patients were elevated in period 1 compared to those in period 3 (Fig. 1). Kidney biopsy in patients with diabetes is typically performed upon referral by a diabetologist for renal dysfunction. During period 1, numerous patients with diabetes were referred to a nephrologist for dialysis. However, in period 3, patients with diabetes were referred to nephrologists at an earlier stage of CKD to consider CKD management. Consequently, Cre values at the time of kidney biopsy in patients with diabetes decreased. Three patterns of renal involvement are observed in patients with diabetes: typical DN, nondiabetic renal involvement, and a combination of the two. Kidney biopsies were performed to differentiate between these three patterns [7, 24].

The prevalence of BNS among all kidney biopsies, including those of patients without ESKD at our institution, provides additional perspectives. During the observation period from 1985 to 2018, 6763 kidney biopsies were performed at our institution. Among these, 271 (4.0%) were diagnosed with BNS or nephrosclerosis, with no significant findings other than atherosclerosis. Among these 271 patients, one patient progressed to ESKD. Furthermore, the present analysis was based on the cases that had undergone kidney biopsy. Consequently, renal histology in cases where kidney biopsy could not be performed due to the small kidney size remains unknown. Such cases may include BNS. Sumida et al. investigated the clinical picture of BNS in a large number of cases using the J-RBR. They found that BNS was more prevalent in elderly patients with preserved renal function and low proteinuria levels. In our investigation of dialysis cases without kidney biopsy, we observed that the pathogenesis of dialysis in such patients is often influenced by extrarenal lesions, such as systemic atherosclerotic diseases, including cardiac and hepatic conditions, rather than by a gradual decline in renal function. Moreover, certain diseases can be diagnosed without kidney biopsy in cases where the procedure cannot be performed. These include ADPKD, SLE, AAV, AMY, pyelonephritis and genetic disorders such as Alport. In patients with renal failure complicated by DM, the typical histology of diabetic nephropathy can be inferred in cases of diabetic retinopathy; however, it is challenging to assess cases without retinopathy. Kwon provides a valuable reference in this regard [24]. The results of kidney biopsies in patients with diabetes revealed that diabetic nephropathy was diagnosed in 37% of cases, kidney diseases other than diabetic nephropathy in 54.3%, and complications in 8.5%.

The strength of this cohort study lies in its extensive follow-up period of 35 years, which facilitated the identification of patients who ultimately developed ESKD or who initiated dialysis. Among the diverse histological diagnoses, we were able to ascertain the kidney diseases listed in Table 1 as the causes of ESKD, indicating that these histological diagnoses at the time of kidney biopsy represent a potential risk factor for subsequent ESKD development.

The limitations of this study include its single-center design, which may introduce bias for kidney biopsy, although the indication for kidney biopsy was consistent for all patients regardless of the presence of DM, and a multicenter study is necessary to obtain a comprehensive representation of the situation. In addition, it is noteworthy that the renal tissue of patients with ESKD who presented to the hospital with atrophic kidneys were not examined, as these patients are not typically evaluated by kidney biopsy. Furthermore, it is often challenging to establish a diagnosis from kidney biopsy in patients with numerous sclerotic glomeruli and very few remaining functioning glomeruli, and the validity of the diagnosis of FGS in a few segmental sclerotic glomeruli in the absence of predominant deposition on IF or EM is questionable. Lastly, the cohort did not include patients who did not required dialysis; therefore, the interpretation of the data should take this limitation into account.

In conclusion, given that the criteria for kidney biopsy were consistent for patients with and without DM at our institution, we posit that the findings of our study provide a comprehensive representation of the nephropathy types that contribute to ESKD.

## Supplementary Information

Below is the link to the electronic supplementary material.

Supplementary file1 (DOCX 37 KB)

## Data Availability

The data underlying this study will be made available upon reasonable request by the corresponding author.

## Abbreviations

AA-Amy: Amyloid A amyloidosis
AAV: ANCA-associated vasculitis
ANCA: Antineutrophil cytoplasmic autoantibody
anti-GBM: Anti-glomerular basement membrane disease
BNS: Benign nephrosclerosis
β2MG: Beta-2 microglobulin
CCE: Cholesterol crystal embolism
Cryo: Cryoglobulinemia
DM: Diabetes mellitus
DN: Diabetic nephropathy
EDDs: Electron-dense deposits
EM: Electron microscopy
ELISA: Enzyme-linked immunosorbent assay
ESKD: End-stage kidney disease
FGS: Focal segmental glomerulosclerosis
GBM: Glomerular basement membrane
Gout: Gouty nephropathy
HC-Amy: Heavy-chain amyloidosis
IgAN: IgA nephropathy
IgAV: IgA vasculitis
IF: Immunofluorescence microscopy
ING: Idiopathic nodular glomerulosclerosis
ITG: Immunotactoid (microtubular) glomerulopathy
LC-Amy: Light-chain amyloidosis
LM: Light microscopy
LN: Lupus nephritis
MCD: Minimal change disease
MGN: Membranous glomerulonephritis
Mit: Mitochondrial nephropathy
MNS: Malignant nephrosclerosis
MPGN: Membranoproliferative glomerulonephritis
MPO: Myeloperoxidase
OFD-1: Orofaciodigital syndrome 1 nephropathy
PR3: Leukocyte proteinase 3
SRC: Scleroderma renal crisis
TIN: Tubulointerstitial nephritis
TMA: Thrombotic microangiopathy
UMOD: Uromodulin nephropathy

## References

[1] Nitta K, Goto S, Masakane I, Hanafusa N, Taniguchi M, Hasegawa T, et al. Annual dialysis data report for 2018, JSDT Renal Data Registry: survey methods, facility data, incidence, prevalence, and mortality. Renal Replacement Therapy. 2020;6(1):41. 10.1186/s41100-020-00286-9.

[2] Collins AJ, Foley RN, Chavers B, Gilbertson D, Herzog C, Johansen K, et al. ’United States Renal Data System 2011 Annual Data Report: Atlas of chronic kidney disease & end-stage renal disease in the United States. Am J Kidney Dis. 2012;59(1):e1-420. 10.1053/j.ajkd.2011.11.015. 10.1053/j.ajkd.2011.11.015 22177944

[3] Navaratnarajah A, Sambasivan K, Cook TH, Pusey C, Roufosse C, Willicombe M. Predicting long-term renal and patient survival by clinicopathological features in elderly patients undergoing a renal biopsy in a UK cohort. Clin Kidney J. 2019;12(4):512–20. 10.1093/ckj/sfz006. 31384442 10.1093/ckj/sfz006 PMC6672059

[4] Matsuo S, Imai E, Horio M, Yasuda Y, Tomita K, Nitta K, et al. Revised equations for estimated GFR from serum creatinine in Japan. Am J Kidney Dis. 2009;53(6):982–92. 10.1053/j.ajkd.2008.12.034. 19339088 10.1053/j.ajkd.2008.12.034

[5] Hiramatsu R, Hoshino J, Suwabe T, Sumida K, Hasegawa E, Yamanouchi M, et al. Membranoproliferative glomerulonephritis and circulating cryoglobulins. Clin Exp Nephrol. 2014;18(1):88–94. 10.1007/s10157-013-0810-z. 23722669 10.1007/s10157-013-0810-z PMC3923107

[6] Tervaert TW, Mooyaart AL, Amann K, Cohen AH, Cook HT, Drachenberg CB, et al. Pathologic classification of diabetic nephropathy. J Am Soc Nephrol. 2010;21(4):556–63. 10.1681/asn.2010010010. 20167701 10.1681/ASN.2010010010

[7] Ubara Y, Kawaguchi T, Nagasawa T, Miura K, Katsuno T, Morikawa T, et al. Kidney biopsy guidebook 2020 in Japan. Clin Exp Nephrol. 2021;25(4):325–64. 10.1007/s10157-020-01986-6. 33606126 10.1007/s10157-020-01986-6 PMC7966701

[8] Mazzucco G, Bertani T, Fortunato M, Bernardi M, Leutner M, Boldorini R, et al. Different patterns of renal damage in type 2 diabetes mellitus: a multicentric study on 393 biopsies. Am J Kidney Dis. 2002;39(4):713–20. 10.1053/ajkd.2002.31988. 11920336 10.1053/ajkd.2002.31988

[9] Sharma SG, Bomback AS, Radhakrishnan J, Herlitz LC, Stokes MB, Markowitz GS, et al. The modern spectrum of renal biopsy findings in patients with diabetes. Clin J Am Soc Nephrol CJASN. 2013;8(10):1718–24. 10.2215/cjn.02510213. 23886566 10.2215/CJN.02510213 PMC3789339

[10] Hsu CY. Does non-malignant hypertension cause renal insufficiency? Evidence-based perspective. Curr Opin Nephrol Hypertens. 2002;11(3):267–72. 10.1097/00041552-200205000-00001. 11981255 10.1097/00041552-200205000-00001

[11] Freedman BI, Iskandar SS, Appel RG. The link between hypertension and nephrosclerosis. Am J Kidney Dis. 1995;25(2):207–21. 10.1016/0272-6386(95)90001-2. 7847347 10.1016/0272-6386(95)90001-2

[12] Sumida K, Hoshino J, Ueno T, Mise K, Hayami N, Suwabe T, et al. Effect of proteinuria and glomerular filtration rate on renal outcome in patients with biopsy-proven benign nephrosclerosis. PLoS ONE. 2016;11(1): e0147690. 10.1371/journal.pone.0147690. 26809068 10.1371/journal.pone.0147690 PMC4726632

[13] Hsu CY. Does treatment of non-malignant hypertension reduce the incidence of renal dysfunction? A meta-analysis of 10 randomised, controlled trials. J Hum Hypertens. 2001;15(2):99–106. 10.1038/sj.jhh.1001128. 11317188 10.1038/sj.jhh.1001128

[14] Appel LJ, Wright JT Jr, Greene T, Agodoa LY, Astor BC, Bakris GL, et al. Intensive blood-pressure control in hypertensive chronic kidney disease. N Engl J Med. 2010;363(10):918–29. 10.1056/NEJMoa0910975. 20818902 10.1056/NEJMoa0910975 PMC3662974

[15] Caetano ER, Zatz R, Saldanha LB, Praxedes JN. Hypertensive nephrosclerosis as a relevant cause of chronic renal failure. Hypertension. 2001;38(2):171–6. 10.1161/01.hyp.38.2.171. 11509471 10.1161/01.hyp.38.2.171

[16] Vikse BE, Aasarød K, Bostad L, Iversen BM. Clinical prognostic factors in biopsy-proven benign nephrosclerosis. Nephrol Dial Transplant. 2003;18(3):517–23. 10.1093/ndt/18.3.517. 12584273 10.1093/ndt/18.3.517

[17] De Vriese AS, Sethi S, Nath KA, Glassock RJ, Fervenza FC. Differentiating primary, genetic, and secondary FSGS in adults: a clinicopathologic approach. J Am Soc Nephrol. 2018;29(3):759–74. 10.1681/asn.2017090958. 29321142 10.1681/ASN.2017090958 PMC5827609

[18] D’Agati VD, Fogo AB, Bruijn JA, Jennette JC. Pathologic classification of focal segmental glomerulosclerosis: a working proposal. Am J Kidney Dis. 2004;43(2):368–82. 10.1053/j.ajkd.2003.10.024. 14750104 10.1053/j.ajkd.2003.10.024

[19] Yokoyama H, Sugiyama H, Sato H, Taguchi T, Nagata M, Matsuo S, et al. Renal disease in the elderly and the very elderly Japanese: analysis of the Japan Renal Biopsy Registry (J-RBR). Clin Exp Nephrol. 2012;16(6):903–20. 10.1007/s10157-012-0673-8. 23053590 10.1007/s10157-012-0673-8

[20] Ozeki T, Maruyama S, Nagata M, Shimizu A, Sugiyama H, Sato H, et al. The revised version 2018 of the nationwide web-based registry system for kidney diseases in Japan: Japan Renal Biopsy Registry and Japan Kidney Disease Registry. Clin Exp Nephrol. 2020;24(11):1058–68. 10.1007/s10157-020-01932-6. 32761468 10.1007/s10157-020-01932-6 PMC7524691

[21] Goto K, Imaizumi T, Hamada R, Ishikura K, Kosugi T, Narita I, et al. Renal pathology in adult and paediatric population of Japan: review of the Japan renal biopsy registry database from 2007 to 2017. J Nephrol. 2023;36(8):2257–67. 10.1007/s40620-023-01687-9. 37597092 10.1007/s40620-023-01687-9 PMC10638177

[22] Sumida K, Takeda A, Furuichi K, Uesugi N, Ubara Y, Sato H, et al. Clinicopathological discordance in biopsy-proven nephrosclerosis: a nationwide cross-sectional study of the Japan Renal Biopsy Registry (J-RBR). Clin Exp Nephrol. 2022;26(4):325–32. 10.1007/s10157-021-02161-1. 34812966 10.1007/s10157-021-02161-1 PMC8930907

[23] Shima N, Hayami N, Mizuno H, Kawada M, Sekine A, Sumida K, et al. Arteriovenous fistula-related renal bleeding 5 days after percutaneous renal biopsy. CEN Case Rep. 2019;8(4):280–4. 10.1007/s13730-019-00408-1. 31214889 10.1007/s13730-019-00408-1 PMC6820813

[24] Kwon AG, Sawaf H, Portalatin G, Shettigar S, Herlitz LC, Shafi T, et al. Kidney biopsy findings among patients with diabetes in the cleveland clinic kidney biopsy epidemiology project. Kidney Med. 2024;6(10): 100889. 10.1016/j.xkme.2024.100889. 39310117 10.1016/j.xkme.2024.100889 PMC11414546

