# Supplementary material for: Clinicopathological cohort study of kidney biopsy findings resulting in dialysis during long-term follow-up exceeding 30 years
Source: Clin Exp Nephrol. 2025 May 28;29(11):1637–52. doi: 10.1007/s10157-025-02706-8 (PMC12568825; doi:10.1007/s10157-025-02706-8)
Supplement: Supplementary file 1 — Supplementary file1 (DOCX 37 KB) [file 10157_2025_2706_MOESM1_ESM.docx]

**Supplementary Material**

**Diagnostic features for the respective kidney diseases**

Concise diagnostic features for the respective kidney diseases are as follows. DN was diagnosed in patients with a history of type 1 or type 2 DM when LM revealed mesangial matrix expansion, nodular structure, capsular drop, and arteriolar hyalinosis; IF revealed linear staining of IgG along the glomerular basement membranes (GBMs); and EM showed GBM thickening according to Tervaert’s pathologic classification of DN[1]. IgA nephropathy (IgAN) was diagnosed when LM showed mesangial hypercellularity; IF showed granular staining of IgA in the mesangial area; and EM showed electron-dense deposits (EDDs) in the mesangial area[2]. Focal segmental glomerulosclerosis (FGS) was diagnosed in patients with proteinuria and/or renal dysfunction when LM showed sclerosis (or scarring) in only a small portion of glomeruli, and/or IF showed positive staining of IgM in a part of the glomerulus, and/or EM showed foot process effacement of podocytes and no EDD[3]. Antineutrophil cytoplasmic autoantibody (ANCA)-associated vasculitis (AAV) was diagnosed by crescentic necrotizing glomerulonephritis and/or small artery vasculitis and by an elevated serum myeloperoxidase (MPO) or leukocyte proteinase 3 (PR3)-ANCA titer by enzyme-linked immunosorbent assay (ELISA) [4]. Membranous glomerulonephritis (MGN) was diagnosed when LM showed bubbling/spike appearance or when IF showed granular deposition of IgG along GBMs and EM showed subepithelial EDDs in GBMs. Lupus nephritis (LN) was diagnosed when LM showed each of class I to V[5]; IF showed granular deposition on glomeruli; EM showed EDD on glomeruli; and clinical and immunological criteria specific to systemic lupus erythematosus were fulfilled[5, 6]. Amyloid A amyloidosis (AA-Amy) was diagnosed when amorphous deposits in glomeruli and small arteries were positive for Congo red and apple-green birefringence under polarizing light; immunohistological analysis was positive for AA but negative for AL (κ/λ), β2MG, and transthyretin; and EM showed randomly arranged fibrils of 7 to 12 nm in diameter. Light-chain amyloidosis (LC-Amy) was diagnosed when immunohistological analysis was positive for only LC (κ or λ) and LM and EM showed AA-Amy features. Heavy-chain amyloidosis (HC-Amy) was diagnosed when IF was positive for only IgG and LM and EM showed AA-Amy features. Membranoproliferative glomerulonephritis (MPGN) was diagnosed when LM showed increased mesangial cellularity or endocapillary glomerulonephritis, IF showed IgG staining on the mesangium and/or along the GBMs, and EM showed EDD on the mesangium and/or endothelial space. Malignant nephrosclerosis (MNS) was diagnosed when small arteries showed intimal thickening, fibrinoid necrosis, or thrombosis in the presence of a clinical finding of malignant hypertension with a diastolic blood pressure greater than 130 mm Hg. Tubulointerstitial nephritis (TIN) was diagnosed by the presence of interstitial edema and inflammatory infiltration with various white blood cells without glomeruli being affected. Alport syndrome (Alport) was diagnosed when LM showed FGS or intact glomeruli, but EM showed a basket appearance of the GBM due to thinning and genetic tests showed an X-linked pattern due to mutations in the *COL4A5* gene or autosomal dominant pattern due to mutations in *COL4A3/A4*. Cryoglobulinemia (Cryo) was diagnosed when LM showed endocapillary glomerulonephritis with thrombus-like deposits and IF showed IgM staining on glomeruli. Anti-GBM disease (anti-GBM) was diagnosed when LM showed crescentic necrotizing glomerulonephritis, IF showed linear staining along the GBM, and ELISA showed elevated serum anti-GBM antibodies. Mitochondrial nephropathy (Mit) was diagnosed when LM showed FGS and genetic tests showed a mitochondrial DNA (mtDNA) point mutation encoding mtRNA. Minimal change disease (MCD) was diagnosed when LM showed intact glomeruli, IF showed no immunoglobulin or C3 staining, and EM showed foot process effacement. Idiopathic nodular glomerulosclerosis (ING) was diagnosed when LM showed a multinodular structure consistent with DN and the patient had not history of DM. IgA vasculitis (IgAV) was diagnosed when kidney biopsy showed IgAN and skin purpura biopsy showed leukocytoclastic vasculitis with IgA-positive staining. Immunotactoid glomerulopathy (ITG) was diagnosed when EM showed parallel microtubular deposits with a mean diameter of 31 nm. Orofaciodigital syndrome 1 nephropathy (OFD-1) was diagnosed when LM showed glomerulocystic kidney disease and genetic tests showed mutation of *OFD-1*. Uromodulin nephropathy (UMOD) was diagnosed when LM showed tubulointerstitial fibrosis and genetic tests showed *UMOD* mutation. Gouty nephropathy (Gout) was diagnosed when LM showed uric acid crystals in tubules and the patient had a history of a gouty attack. Scleroderma renal crisis (SRC) is a renal disorder in patients with systemic sclerosis and shows kidney findings similar to MNS. Renal disease caused by cholesterol crystal embolism (CCE) was diagnosed by the existence of atherosclerotic plaques (cleft) in renal small arteries. ANCA negative crescentic glomerulonephritis (ANCA-neg) was diagnosed when crescentic necrotizing glomerulonephritis was detected but ELISA showed a negative serum ANCA value. Thrombotic microangiopathy (TMA) was diagnosed in patients with glomerular endothelial cell disorder with no apparent underlying disease. Obesity nephropathy (Obesity) was diagnosed when the perihilar type of FGS was detected in a patient with a body mass index greater than 30.

Benign nephrosclerosis (BNS) was diagnosed in patients with arteriolosclerosis or arteriosclerosis accompanied by intimal thickening of arteries or arteriolar hyalinosis and preserved intact glomeruli or ischemic collapse of glomeruli or focal global glomerulosclerosis[7, 8] after the above-mentioned diseases were excluded. When two or more of the above diagnoses were present simultaneously, priority was given to conditions that may be directly related to kidney failure.

**The underlying kidney diseases in other dialysis patients and prevalence of smoking and hypertension**

Anticipated renal diseases diagnosed without kidney biopsy were collected from medical records.

**Comprehensive characterization of patients with IgAN, including clinical and histological grades at kidney biopsy**

Renal samples from 86 patients with IgAN were available for the re-evaluation of renal histology according to the criteria described elsewhere.[2, 9, 10] We stratified IgAN patients into two groups based on the biopsy year (before or after 2000) (Fig 4a), age (Fig 4b), proteinuria (Fig 4c), urinary blood level (Fig 4d), and eGFR (Fig 4e), and compared. The median of each factor was chosen to divide the patients into two groups.

**References**:

1. Tervaert TW, Mooyaart AL, Amann K, Cohen AH, Cook HT, Drachenberg CB et al. Pathologic classification of diabetic nephropathy. Journal of the American Society of Nephrology : JASN. 2010;21(4):556-63. doi:10.1681/asn.2010010010.

2. Cattran DC, Coppo R, Cook HT, Feehally J, Roberts IS, Troyanov S et al. The Oxford classification of IgA nephropathy: rationale, clinicopathological correlations, and classification. Kidney international. 2009;76(5):534-45. doi:10.1038/ki.2009.243.

3. D'Agati VD, Fogo AB, Bruijn JA, Jennette JC. Pathologic classification of focal segmental glomerulosclerosis: a working proposal. American journal of kidney diseases : the official journal of the National Kidney Foundation. 2004;43(2):368-82. doi:10.1053/j.ajkd.2003.10.024.

4. Jennette JC, Falk RJ, Bacon PA, Basu N, Cid MC, Ferrario F et al. 2012 revised International Chapel Hill Consensus Conference Nomenclature of Vasculitides. Arthritis and rheumatism. 2013;65(1):1-11. doi:10.1002/art.37715.

5. Weening JJ, D'Agati VD, Schwartz MM, Seshan SV, Alpers CE, Appel GB et al. The classification of glomerulonephritis in systemic lupus erythematosus revisited. Kidney international. 2004;65(2):521-30. doi:10.1111/j.1523-1755.2004.00443.x.

6. Petri M, Orbai AM, Alarcón GS, Gordon C, Merrill JT, Fortin PR et al. Derivation and validation of the Systemic Lupus International Collaborating Clinics classification criteria for systemic lupus erythematosus. Arthritis and rheumatism. 2012;64(8):2677-86. doi:10.1002/art.34473.

7. Sumida K, Hoshino J, Ueno T, Mise K, Hayami N, Suwabe T et al. Effect of Proteinuria and Glomerular Filtration Rate on Renal Outcome in Patients with Biopsy-Proven Benign Nephrosclerosis. PloS one. 2016;11(1):e0147690. doi:10.1371/journal.pone.0147690.

8. Hommos MS, Glassock RJ, Rule AD. Structural and Functional Changes in Human Kidneys with Healthy Aging. Journal of the American Society of Nephrology : JASN. 2017;28(10):2838-44. doi:10.1681/asn.2017040421.

9. Trimarchi H, Barratt J, Cattran DC, Cook HT, Coppo R, Haas M et al. Oxford Classification of IgA nephropathy 2016: an update from the IgA Nephropathy Classification Working Group. Kidney international. 2017;91(5):1014-21. doi:10.1016/j.kint.2017.02.003.

10. Roberts IS, Cook HT, Troyanov S, Alpers CE, Amore A, Barratt J et al. The Oxford classification of IgA nephropathy: pathology definitions, correlations, and reproducibility. Kidney international. 2009;76(5):546-56. doi:10.1038/ki.2009.168.
